# Patient-Level Cancer Prediction Models From a Nationwide Patient Cohort: Model Development and Validation

**DOI:** 10.2196/29807

**Published:** 2021-08-30

**Authors:** Eunsaem Lee, Se Young Jung, Hyung Ju Hwang, Jaewoo Jung

**Affiliations:** 1 Department of Mathematics Pohang University of Science and Technology Pohang-si Republic of Korea; 2 Office of eHealth Research and Businesses Seoul National University Bundang Hospital Seongnam-si Republic of Korea; 3 AMSquare Corporation Pohang-si Republic of Korea

**Keywords:** prediction, model, claim data, cancer, machine learning, development, cohort, validation, database, algorithm

## Abstract

**Background:**

Nationwide population-based cohorts provide a new opportunity to build automated risk prediction models at the patient level, and claim data are one of the more useful resources to this end. To avoid unnecessary diagnostic intervention after cancer screening tests, patient-level prediction models should be developed.

**Objective:**

We aimed to develop cancer prediction models using nationwide claim databases with machine learning algorithms, which are explainable and easily applicable in real-world environments.

**Methods:**

As source data, we used the Korean National Insurance System Database. Every Korean in ≥40 years old undergoes a national health checkup every 2 years. We gathered all variables from the database including demographic information, basic laboratory values, anthropometric values, and previous medical history. We applied conventional logistic regression methods, light gradient boosting methods, neural networks, survival analysis, and one-class embedding classifier methods to effectively analyze high dimension data based on deep learning–based anomaly detection. Performance was measured with area under the curve and area under precision recall curve. We validated our models externally with a health checkup database from a tertiary hospital.

**Results:**

The one-class embedding classifier model received the highest area under the curve scores with values of 0.868, 0.849, 0.798, 0.746, 0.800, 0.749, and 0.790 for liver, lung, colorectal, pancreatic, gastric, breast, and cervical cancers, respectively. For area under precision recall curve, the light gradient boosting models had the highest score with values of 0.383, 0.401, 0.387, 0.300, 0.385, 0.357, and 0.296 for liver, lung, colorectal, pancreatic, gastric, breast, and cervical cancers, respectively.

**Conclusions:**

Our results show that it is possible to easily develop applicable cancer prediction models with nationwide claim data using machine learning. The 7 models showed acceptable performances and explainability, and thus can be distributed easily in real-world environments.

## Introduction

Cancer is a major cause of death, accounting for nearly 10 million deaths worldwide in 2020 [1]. It is a preventable disease requiring major lifestyle modifications [2], for which screening is important because it can help health care professionals with early detection and treatment of several types of cancer before they become aggravated [3]. In the early stages, cancer is normally indolent and symptomless. Thus, nationwide cancer screening programs for the general population have been adopted in many countries [4-8]. A national cancer control program (NCCP) framework, a public health program designed to mitigate the number of cancer cases and deaths and improve quality of life of patients, was proposed by the World Health Organization [6,9]. In South Korea, the NCCP was designed in 1996 and implemented in 1999 to provide free screening services for low-income Medical Aid patients. Beginning in 2000, the NCCP has expanded its target population to include all National Health Insurance (NHI) recipients. Since that time, the survival rate of cancer patients has continued to improve. According to cancer registration statistics in 2013, the relative survival rate of cancer patients has increased to 70.3% [10]. For 7 major cancer, namely, stomach, colorectal, breast, lung, cervical, pancreas, and liver cancer, every NHI beneficiary receives cancer screening tests mainly based on his or her age and gender. For instance, everyone ≥40 years old is examined by upper gastrointestinography or gastrointestinal endoscopy every 2 years to screen for stomach cancer. However, concerns have been raised about this one-size-fits-all cancer screening program because every procedure for cancer screening has its own risks for false-positive cases. For instance, false-positive cases of mammograms for screening breast cancer have resulted in many unnecessary invasive breast excisional biopsies, which reduce the quality of life in women [11,12]. Thus, personalized cancer screening protocols based on patient’s individual risks have been in need since the NCCP was introduced [13,14]. The National Health Insurance System (NHIS) has collected health checkup data since 2003 under a structured data format and made it available for researchers [15]. There are two types of NHIS cohort data: a 1-million-person cohort sampled randomly from all NHI beneficiaries reflecting general characteristics of the entire South Korean population and a 500-thousand-person cohort sampled from those who received national health checkup services. All data include every diagnosis code and medications of each patient in all hospitals and clinics. For beneficiaries of national health checkup services, data include basic anthropometric measurements, laboratory values, past medical history, and family history. Despite the limited number of variables for the development of machine learning algorithms compared to electronic health records (EHRs) in hospitals, this type of data has the substantial advantages of a well-refined structured format and large sample size [16]. The data structure of the NHIS cohort and the monthly claim data from every EHR in hospitals are the same; therefore, the developed patient-level prediction models can be implemented in any EHR system in South Korea. In this study, we aimed to develop practical patient-level prediction models of 7 major cancers with acceptable performances and explainability, which can be distributed easily in real-world environments.

## Methods

### Data Description

We used an NHIS database to develop our cancer prediction models. The NHIS, a mandatory social insurance system, has collected health screening data at the national population level since the mid-1970s [15]. As this is a centralized system, Korean health screening data can be centralized, while paid health care providers act on a per-service basis [17]. The NHIS database consists of 2 different data sets: a health checkup cohort and a national sample cohort [18]. We used the health checkup cohort in the learning process and included training and internal validation and the remaining national sample cohort for external validation.

The NHIS provides a free health checkup program to all NHI members every 2 years. The health checkup cohort contains a total of 514,866 patients’ health checkup records randomly extracted from health insurance members who have undergone a heath checkup program. The national sample cohort contains about 1 million patient records corresponding to about 2.2% of the Korean population in 2002. This data set was collected by considering demographics, such as population, age, and geographic factors. Both data sets include social and economic eligibility variables, health resource utilization status, description, treatment details, disease type, prescription details, and clinic status. The NHIS data set statistics are presented in Table 1.

**Table 1:** Statistics of the National Health Insurance Service data sets (2002-2013).

Description	Health checkup cohort, n	National sample cohort, n
Hospital	51,920	52,483
Patients	514,866	1,113,656
Prescriptions	83,935,395	83,935,395
Visits	96,534,359	119,362,188
Diagnostic codes (full code name)	17,385	19,626
Diagnostic codes (first 3 digits)	2160	2319
Annual patient visits, mean	15.6	8.9
Diagnostic codes/visit, mean	2.4	2.5
Drug/prescription, mean	4.4	4.4

### Study Population Definition

It is mandatory that all cancer patients in South Korea be enrolled into a national cancer management program in the hospital where the cancer is diagnosed so that cancer patients only pay 5% of the total medical cost [19]. This means that almost all cancer patients in South Korea can be identified by diagnosis codes registered in the NHIS database [20].

We used the Korean Classification of Disease version 7, which is compatible with International Classification of Disease (ICD)-9 and defined the following 7 major cancers [21]: liver cancer (malignant neoplasm of the liver and intrahepatic bile ducts), C22; lung cancer (malignant neoplasm of the bronchus and lung), C34; colorectal cancer (malignant neoplasm of the colon, rectosigmoid junction, and rectum), C18, C19, and C20; pancreatic cancer (malignant neoplasm of the pancreas), C25; stomach cancer (malignant neoplasm of the stomach), C16; and breast cancer (malignant neoplasm of the breast), C50; and cervical cancer (malignant neoplasm of the cervix uteri), C53.

The prevalence of each cancer is presented in Table 2.

**Table 2:** The number of cancer-free patients and the number of cancer patients diagnosed for each cancer.

Patient type	Liver	Lung	Colorectal	Pancreatic	Stomach	Breast	Cervical
Free, n	234,659	233,931	233,203	235,633	232,493	91,982	92,736
Diagnosed, n	1587	2335	2845	551	3679	1029	306

### Input Features and Algorithms

First, we used basic features consisting of simple demographic information, including age and gender, health examination, and survey results (18 features, level 1). Second, we added 11 more features obtained from a questionnaire, including the patient's medical history and family medical history (29 features, level 2). Third, we included 10 specific disease diagnostic records that appeared significant through univariate analysis for each cancer (39 features, level 3). The specific codes for each of the 10 cancers are provided in Multimedia Appendix 1.

**Figure 1:**
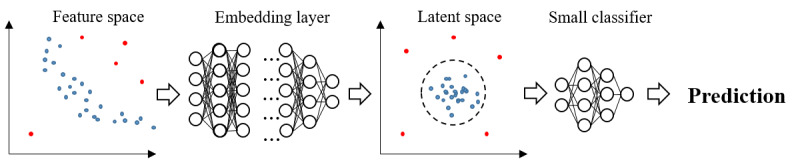
Concept of one-class embedding classifier.

To predict future cancers, we focused on cancer incidence within the next 5 years based on the time of screening. We first trained our predictive model with 4 common machine learning models: logistic regression (LR), random forest (RF), Light Gradient Boosting Machine (LGBM; a tree-based gradient boosting model), and multilayer perceptron (MLP). Further, we built a one-class embedding classifier (OCEC), which is a deep anomaly detection-based model (Figure 1). This method assumes that the data have one large class and several types of small anomalies not included in that class. This is an appropriate assumption because, while most people have normal screening records, few have cancer. To build our OCEC structure, we modified a deep one-class classification, the first deep learning–based anomaly detection model [22]. We then added a small classifier to the latent space to predict future cancer. The hyperparameters used for training models are shown in Multimedia Appendix 2.

### Model Evaluation Strategy

We divided an entire health checkup cohort, with 80% placed into a training set and 20% placed into a validation set. The model was trained only with the training set while the internal validation set was not used in the learning process. After training, the model output a prediction score for the probability of developing cancer in the next 5 years after the input year.

A cancer prediction problem is heavily imbalanced because the proportion of cancer-diagnosed patients is too small. In our data, the proportions of cancer-diagnosed patients were <2% for all 7 cancers. Thus, we used the area under the receiver operating characteristic curve (AUROC) and area under the precision recall curve (AUPRC) score to evaluate our models. The AUROC is an evaluation metric with values between 0 and 1 that is widely used as an evaluation metric for the imbalance problem, while the AUPRC combines recall and precision and corresponds to the average of the precision according to the precision recall curve. The baseline for AUROC is always 0.5, meaning a random classifier would produce an AUROC of 0.5. However, with AUPRC, the baseline is equal to the fraction of positive cancer cases (number of positive examples/total number of examples). The baseline AUPRC for each cancer in both the internal and external validation sets is shown in Table 3.

**Table 3:** The baseline area under the precision recall curve for the internal and external validation sets.

Validation set	Liver	Lung	Colorectal	Pancreatic	Stomach	Breast	Cervical
Internal validation	4.45×10^–3^	6.03×10^–3^	7.72×10^–3^	1.50×10^–3^	1.04×10^–2^	7.65×10^–3^	2.39×10^–3^
External validation	2.96×10^–3^	3.86×10^–3^	5.52×10^–3^	1.01×10^–3^	6.65×10^–3^	7.97×10^–3^	2.22×10^–3^

**Figure 2:**
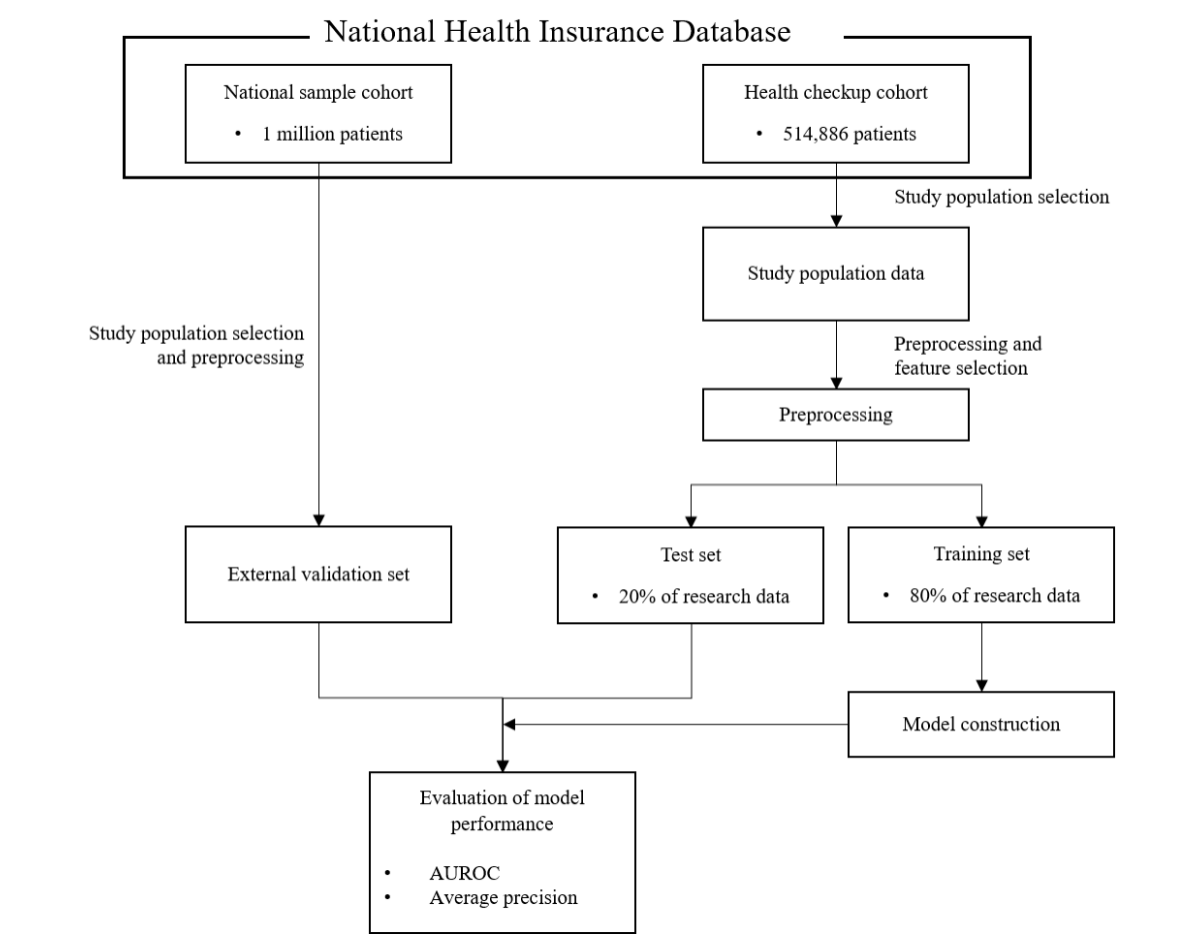
Flow chart of the overall process. AUROC: area under the receiver operating characteristic curve.

We evaluated the above metrics for both internal and external validation sets and compared the results. Additionally, for the external data set, we used the survival analysis method. We plotted Kaplan-Meier cumulative density curves to see the actual effectiveness of the predictive score. The study flow chart for learning and verification of the overall process is shown in Figure 2.

The NHIS institutional review board approved all data requests for research purposes (NHIS-2017-2-326). Because this public database is fully anonymized, institutional approval of Seoul National University Bundang Hospital (SNUBH) was waived by the institutional review board (X-2009-634-902).

## Results

### Performance of Cancer Prediction Models

Table 4 shows the internal validation results for each cancer across the 5 models. Overall, the LGBM and deep learning models performed better than did LR and RF. The former models performed well in terms of AUROC and AUPRC scores. LR, the most widely used classic model, showed low AUPRC scores, while RF had a low AUROC.

Notably, more than half of the OCEC AUROC scores were top rated compared to other models. Two models, OCEC and MLP, are both deep learning structured models. However, OCEC uses dense dimension reduction and performed better for both AUROC and AUPRC score compared to the MLP model. This shows that the anomaly-based one-class classification model can be a suitable deep learning structure for rare disease prediction.

When looking at the internal validation results of each cancer, liver and lung cancers showed the best results (AUROC>0.8), followed by stomach, pancreatic, and colorectal cancers (0.8>AUROC>0.7). Cervical and breast cancers (both female cancers) showed the lowest results (0.7>AUROC>0.6). The same findings also appeared in the external validation (Table 5).

According to feature level, the results tended to improve as feature level increased from level 1 to 3, but this was not significant. However, in some cases, the opposite tendency was observed.

The findings for the external validation score were similar to those of the internal score. Interestingly, the external validation scores (Table 5) were higher than the internal ones overall.

**Table 4:** Internal validation performance of outcome prediction across models.

Cancer type	Feature level	LGBM^a^			LR^b^			RF^c^			MLP^d^			OCEC^e^	
		AUROC^f^	AUPRC^g^	AUROC		AUPRC	AUROC		AUPRC	AUROC		AUPRC	AUROC		AUPRC
**Liver**															
	Level 1	0.858	0.359	0.836		0.045	0.748		0.359	0.858		0.296	0.857		0.313
	Level 2	0.868	0.363	0.841		0.048	0.770		0.342	0.856		0.297	0.860		0.301
	Level 3	0.871	0.383	0.852		0.080	0.788		0.361	0.860		0.315	0.868		0.334
**Lung**															
	Level 1	0.845	0.396	0.823		0.106	0.735		0.366	0.845		0.360	0.849		0.382
	Level 2	0.845	0.395	0.822		0.110	0.750		0.366	0.832		0.338	0.841		0.338
	Level 3	0.845	0.401	0.829		0.130	0.754		0.367	0.841		0.345	0.843		0.343
**Colorectal**															
	Level 1	0.790	0.385	0.764		0.055	0.707		0.366	0.794		0.347	0.795		0.371
	Level 2	0.792	0.387	0.767		0.063	0.701		0.363	0.790		0.321	0.798		0.342
	Level 3	0.794	0.385	0.769		0.075	0.704		0.360	0.791		0.322	0.796		0.342
**Pancreatic**															
	Level 1	0.723	0.300	0.724		0.017	0.676		0.316	0.744		0.234	0.746		0.259
	Level 2	0.720	0.281	0.727		0.018	0.669		0.309	0.725		0.240	0.745		0.240
	Level 3	0.723	0.271	0.730		0.018	0.682		0.311	0.730		0.225	0.743		0.231
**Stomach**															
	Level 1	0.787	0.385	0.768		0.086	0.713		0.353	0.793		0.348	0.798		0.367
	Level 2	0.790	0.382	0.770		0.092	0.704		0.351	0.796		0.345	0.800		0.345
	Level 3	0.791	0.383	0.772		0.108	0.715		0.351	0.787		0.329	0.795		0.329
**Breast**															
	Level 1	0.684	0.344	0.689		0.077	0.666		0.343	0.705		0.325	0.713		0.332
	Level 2	0.696	0.345	0.696		0.083	0.681		0.346	0.706		0.324	0.711		0.327
	Level 3	0.722	0.357	0.733		0.129	0.689		0.353	0.734		0.339	0.749		0.345
**Cervical**															
	Level 1	0.647	0.268	0.667		0.013	0.656		0.273	0.671		0.263	0.690		0.265
	Level 2	0.672	0.271	0.669		0.012	0.632		0.274	0.660		0.266	0.670		0.266
	Level 3	0.653	0.296	0.612		0.027	0.679		0.301	0.638		0.275	0.645		0.279

^a^LGBM: Light Gradient Boosting Model.

^b^LR: logistic regression.

^c^RF: random forest.

^d^MLP: multilayer perceptron.

^e^OCEC: one-class embedding classifier.

^f^AUROC: area under receiver operator characteristics curve.

^g^AUPRC: area under precision recall curve.

**Table 5:** External performance of outcome prediction across models.

Cancer type	Feature level	LGBM^a^		LR^b^			RF^c^			MLP^d^			OCEC^e^	
		AUROC^f^	AUPRC^g^	AUROC	AUPRC	AUROC		AUPRC	AUROC		AUPRC	AUROC		AUPRC
**Liver**														
	Level 1	0.910	0.485	0.893	0.065	0.815		0.502	0.911		0.433	0.912		0.442
	Level 2	0.909	0.485	0.895	0.067	0.826		0.488	0.900		0.391	0.911		0.433
	Level 3	0.915	0.514	0.907	0.120	0.838		0.527	0.910		0.463	0.919		0.471
**Lung**														
	Level 1	0.896	0.465	0.875	0.097	0.789		0.468	0.898		0.431	0.897		0.450
	Level 2	0.895	0.463	0.875	0.104	0.788		0.465	0.886		0.296	0.894		0.401
	Level 3	0.897	0.464	0.879	0.118	0.794		0.471	0.887		0.402	0.894		0.408
**Colorectal**														
	Level 1	0.872	0.455	0.858	0.070	0.776		0.482	0.883		0.426	0.887		0.449
	Level 2	0.874	0.453	0.858	0.076	0.780		0.481	0.874		0.394	0.887		0.423
	Level 3	0.877	0.455	0.859	0.085	0.776		0.473	0.882		0.393	0.884		0.415
**Pancreatic**														
	Level 1	0.891	0.420	0.884	0.029	0.753		0.456	0.898		0.360	0.904		0.336
	Level 2	0.888	0.405	0.884	0.030	0.747		0.450	0.883		0.335	0.902		0.337
	Level 3	0.885	0.407	0.886	0.039	0.759		0.450	0.883		0.323	0.897		0.336
**Stomach**														
	Level 1	0.889	0.481	0.863	0.088	0.795		0.478	0.891		0.457	0.894		0.440
	Level 2	0.891	0.480	0.864	0.095	0.793		0.479	0.887		0.422	0.893		0.436
	Level 3	0.889	0.478	0.864	0.109	0.792		0.473	0.885		0.401	0.890		0.413
**Breast**														
	Level 1	0.763	0.485	0.704	0.108	0.750		0.492	0.686		0.406	0.753		0.421
	Level 2	0.771	0.488	0.716	0.106	0.745		0.492	0.678		0.396	0.697		0.410
	Level 3	0.780	0.497	0.759	0.143	0.757		0.491	0.730		0.411	0.745		0.429
**Cervical**														
	Level 1	0.729	0.364	0.742	0.021	0.722		0.375	0.671		0.293	0.735		0.336
	Level 2	0.721	0.370	0.744	0.018	0.715		0.377	0.710		0.338	0.732		0.334
	Level 3	0.749	0.386	0.760	0.058	0.731		0.400	0.744		0.349	0.744		0.354

^a^LGBM: Light Gradient Boosting Model.

^b^LR: logistic regression.

^c^RF: random forest.

^d^MLP: multilayer perceptron.

^e^OCEC: one-class embedding classifier.

^f^AUROC: area under receiver operator characteristics curve.

^g^AUPRC: area under precision recall curve.

### Survival Analysis

**Figure 3:**
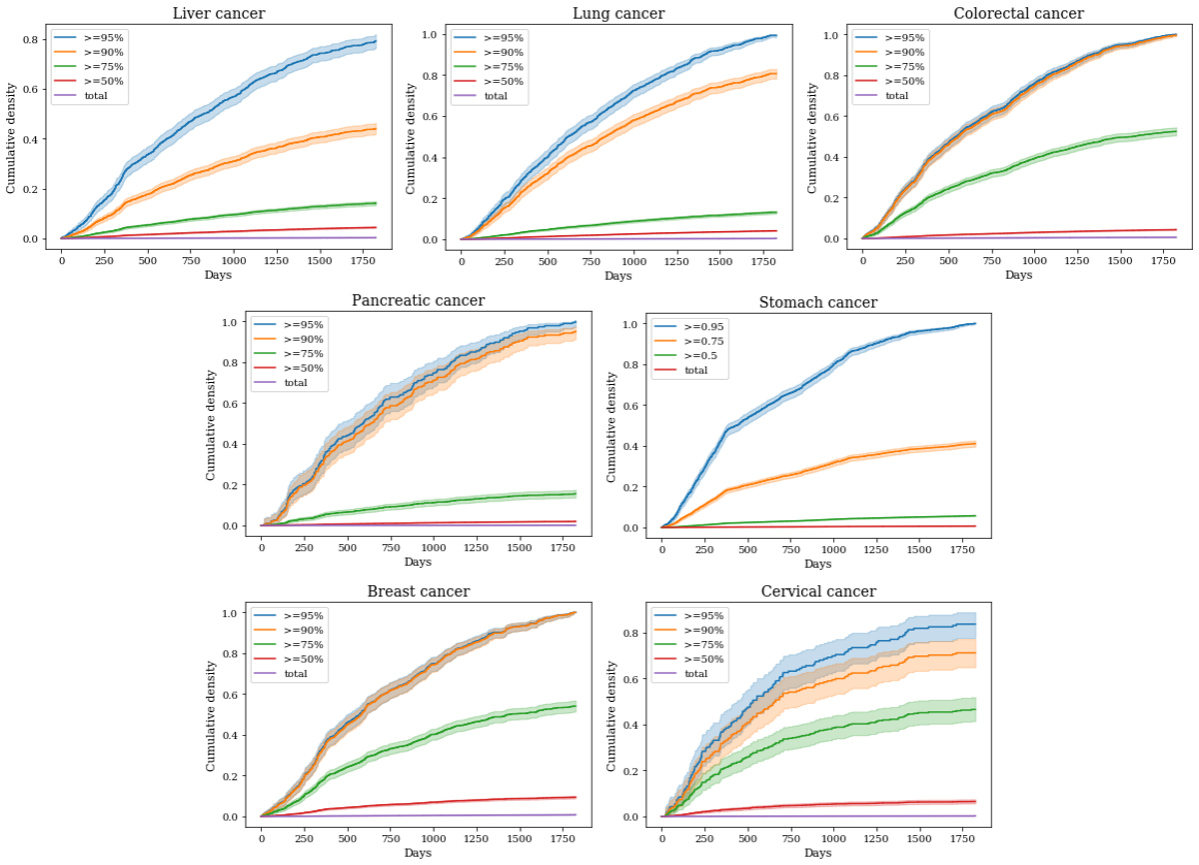
Kaplan-Meier cumulative density curves.

To unveil the actual cancer incidence according to the predicted value, we use a survival analysis method. We analyzed the prediction scores of the LGBM model, one of the best performing of the aforementioned models. The prediction score indicates the probability of developing cancer within 5 years from the screening date. Therefore, the closer the prediction score is to 1, the likelier it is that cancer will actually occur after a certain time. We analyzed 5 groups of patients by prediction scores: group 1 (prediction score ≥0.95), group 2 (prediction score ≥0.90), group 3 (prediction score ≥0.75), group 4 (prediction score ≥0.50), and total patient groups. We drew Kaplan-Meier cumulative density curves for each group and compared them. In Figure 3, the x-axis represents time from the screening date, and the y-axis the rate of cancer incidence within the group. All these analyses were performed with external validation data. As the proportion of cancer patients is <1% for all cancers, the cumulative density curves are attached to the x-axis. The density curve of the group with the higher probability score is located at the higher cumulative density value (y-axis). These trends were collectively observed in all cancers and show the reliability of our models. Significantly, >80% of patients in group 1 actually developed cancer within 5 years.

### Model Explainability

With the LGBM and Shapley Additive Explanations (SHAP) method we can explain how the model outputs cancer prediction scores [23]. We can evaluate which features are the most important to predicting future cancer. Moreover, it is possible to know whether a feature has a positive effect or a negative effect.

Table 6 shows the top 5 features for predicting cancer incidence for each type of cancer. Overall, age was the most important variable as was gender except for women’s cancers. In addition, drinking frequency, alcohol consumption, and total cholesterol levels were all relevant factors.

In particular, aspartate aminotransferase and gamma-glutamyl transferase levels are important for liver cancer. Smoking frequency is an important variable in lung cancer but not in other cancers. Similarly, drinking is the third most important feature for stomach cancer. In breast and pancreatic cancers, blood glucose levels were a more important variable than they were for other cancers. For further details on SHAP values including correlations between each variable and cancer prediction, see Multimedia Appendix 3.

**Table 6:** Top 5 features by Shapley Additive Explanations.

Liver	Lung	Colorectal	Pancreatic	Stomach	Breast	Cervical
Age	Age	Age	Age	Age	Age	Age
GTP^a^	Smoking	Sex	Hemoglobin	Sex	BMI	Fasting glucose
AST^b^	Sex	BMI	Total cholesterol	BMI	Total cholesterol	BMI
Total cholesterol	BMI	Total cholesterol	BP^c^ (high)	Drinking habit	Fasting glucose	Conjunctivitis
BMI	GTP	Fasting glucose	BMI	Hemoglobin	BP (high)	Total cholesterol

^a^GTP: guanosine triphosphate.

^b^AST: aspartate aminotransferase.

^c^BP: blood pressure.

## Discussion

In this study, we used nationwide population-based health care data to construct a machine learning model to predict the future incidence of 7 common types of cancer: liver, stomach, colorectal, lung, pancreatic, breast, and cervical cancer.

Among the 5 distinct models, the LGBM and OCEC, which is our original structure, performed best. Both models had a higher AUROC and AUPRC than did the other models. Interestingly, OCEC scored best in terms of AUROC score and outperformed the normal deep learning method (MLP). Our dense dimension reduction method with one-class anomaly insights was the best model structure.

All models performed well on the external validation set; therefore, it was a success in terms of generalization. Actually, the external validation results were even better than those of the internal validation, thus ensuring the generalizability of our models. We believe that this result was obtained due to the different sampling methods use between the training and validation cohort: the training data set consisted of only those with health checkup information, whereas the validation data set was sampled based on patients' demographic information. As such, the national sample cohort has a similar distribution to the health checkup cohort. In addition, the national sample cohort has a sufficient number of data samples, thus producing good external validation results.

We drew a Kaplan-Meier cumulative density curve for the LGBM model, which is the traditional way to determining whether the marker (prediction score in this case) is suitable to predict cancer occurrence. More than 80% of the people with a prediction score ≥0.95 actually developed cancer within 5 years from the screening date. This is a significant result, which shows that our model can be a powerful tool for identifying high-risk groups. These high-risk groups could then take precautions before the cancer develops. In female cancers, such as breast and cervical cancer, the predictive power was lower than in other cancers. This is probably because both the size of the total female data sample and the number of cancer patients were relatively small. On the other hand, the predictive power for liver and lung cancer was very high. Our data set included liver-related features such as glutamic oxaloacetic transaminase and glutamate pyruvate transaminase. Moreover, we believed that smoking- and drinking-related features also helped predict these cancers. Accordingly, we can conclude that securing high-quality features and a large amount of data can improve predictive power.

There have been previous attempts to develop cancer prediction models with various input features. Japanese researchers developed a prediction model for the 10-year risk of hepatocellular carcinoma in middle-aged Japanese people using data obtained from 17,654 Japanese aged 40 to 69 years who participated in regular health checkups [24]. They obtained a higher AUROC (0.933) than did our models (0.912 in level 1 feature set). However, they did not provide AUPRC, which is important in real-world settings. Furthermore, they used viral markers of hepatitis virus B and C, which are not commonly checked in the normal population. Compared to the previous model, our model used general input features that are easily obtainable, and we acquired a comparable AUROC to the previous model. A Korean research group developed a risk prediction model using Cox proportional hazard regression models for colorectal cancer with a population of 846,559 men and 479,449 women who participated in health examinations by the National Health Insurance Corporation, and they obtained C statistics between 0.69 and 0.78 [25]. They used a similar data set with a different timespan (from 1997 to1997) from our data set and obtained a similar performance to our model (0.730 vs 0.780) This means the performance of classifiers tends to depend on the training data set characteristics rather than the data and time windows. In another study, a multivariable lung cancer risk prediction model including low-dose computed tomography screening results from 22,229 participants obtained an AUROC of 0.761, which is lower than that of our model (0.898 in the MLP model) [26]. Importantly, our model showed a higher performance with an AUROC of 0.875 in a simple linear model (logistic regression with level 1 input features).

In terms of real-world implementation, this study has several implications. Thus far, many studies using machine learning have been conducted on EHR time sequence data. One study aimed to predict heart failure from EHR data [27], and others focused on diabetes development [28-30] or hypertension [31,32]. Furthermore, a few studies have used nationwide claim health checkup data to create a cancer prediction model [33-36]. To solve the overdiagnosis problem of cancer screening programs resulting in unnecessary intervention, accurate, easy-to-implement, patient-level models should be developed. Applying the developed algorithms in previous studies to hospital sites requires considerable effort because the data structure of the developed model differs from that of hospitals. However, our models have the same data structure as the national health care claim data generated on a monthly basis, which means that our models can be directly applied to EHR and makes this study meaningful in terms of its easy applicability. In addition, since we applied an explainable model to LGBM, every doctor can access the modifiable risk factors from the predicted results.

Our research has several limitations. First, this study used only South Korean nationwide claim data. Depending on the country, the performance of the developed algorithms can differ. The value of NHIS data is well-known, and the data have been used in previous epidemiologic studies. Furthermore, we validated the developed algorithms using another database. Future additional external model validations using claim data from other countries can provide robustness to the models. Second, comparative effectiveness research is needed to prove the usefulness of the developed models. Conventional screening models can be compared to new patient-level prediction models in terms of cost and the number of false-positives avoided by the new models.

## Appendix

Multimedia Appendix 1 Ten disease codes used as features for each cancer.

Multimedia Appendix 2 Hyperparameters used for training models.

Multimedia Appendix 3 Shapley Additive Explanations (SHAP) summary plot for each cancer.

## Abbreviations

AUPRC: area under precision recall curve
AUROC: area under receiver operator characteristics curve
EHR: electronic health record
LGBM: Light Gradient Boosting Machine
LR: logistic regression
MLP: multilayer perceptron
NCCP: national cancer control program
NHI: National Health Insurance
NHIS: National Health Insurance System
OCEC: one-class embedding classifier
RF: random forest
SHAP: Shapley Additive Explanations
SNUBH: Seoul National University Bundang Hospital
