# Supplementary material for: Patient-Level Cancer Prediction Models From a Nationwide Patient Cohort: Model Development and Validation
Source: JMIR Med Inform. 2021 Aug 30;9(8):e29807. doi: 10.2196/29807 (PMC8438609; doi:10.2196/29807)
Supplement: Multimedia Appendix 1 [file medinform_v9i8e29807_app1.docx]

**Multimedia Appendix 1. Ten disease codes used as features for each cancer.**

**Table S1. Ten disease codes used as features for each cancer.**

| LIVER | LUNG | COLORECTAL | PANCREATIC | STOMACH | BREAST | CERVICAL |
| --- | --- | --- | --- | --- | --- | --- |
| K741 | J449 | H251 | E118 | N40 | D24 | D069 |
| K746 | J440 | N40 | Z961 | H259 | N63 | N879 |
| B181 | J189 | H250 | H360 | M4806 | N644 | H10 |
| B182 | Z961 | H258 | H258 | I639 | H521 | N72 |
| B18 | N40 | H259 | K053 | H250 | B373 | H258 |
| K739 | J180 | M4806 | E115 | M4786 | N768 | N952 |
| R040 | J45 | K590 | E119 | M480 | J320 | K27 |
| K769 | J42 | M480 | E14 | K257 | J329 | H811 |
| E115 | H251 | E11 | K590 | E119 | D259 | H104 |
| E114 | K590 | I10 | J449 | E11 | N72 | B300 |
