# Supplementary material for: Patient-Level Cancer Prediction Models From a Nationwide Patient Cohort: Model Development and Validation
Source: JMIR Med Inform. 2021 Aug 30;9(8):e29807. doi: 10.2196/29807 (PMC8438609; doi:10.2196/29807)
Supplement: Multimedia Appendix 2 [file medinform_v9i8e29807_app2.docx]

**Multimedia Appendix 2. Hyper-parameters used for training models.**

After experimenting with various values, the following hyper-parameter setting produced the best performance. We implemented algorithms with Tensorflow for MLP and OCEC and Scikit-Learn for Logistic regression, Random Forest.

**Table S2. Hyper-parameter settings for training the models**

| Model | Package | Hyper-parameter |
| --- | --- | --- |
| LightGBM | Lightgbm | max_depth=6, n_estimators=200, class_weight='scale_pos_weight', reg_alpha=0.1 |
| Logistic Regression | scikit-learn/  LogisticRegression | l2 regularization: 0.1 |
| Random Foreset | scikit-learn/  RandomForestCl  assifier | max_depth=5, n_estimators=200 |
| MLP with  breast, cervical cancers | Tensorflow/  sequential model | number of layers: 2, activation: ReLU, hidden_size: (64, 64), l2_regularization: 0.01,   dropout_rate:0.2, optimizer: Adam, loss: binary_cross_entropy |
| MLP without  breast, cervical cancers | Tensorflow/  sequential model | number of layers: 2, hidden_size: (48, 48), activation: ReLU, l2_regularization: 0.01,  dropout_rate:0.2, optimizer: Adam, loss: binary_cross_entropy |
| OCEC with  breast, cervical cancers | Tensorflow/  custom model | number of encoder layers: 4, encoder_hidden_size: (64, 48, 24, 12),  number of classifier layers: 1, classifier_hidden_size: 16,  l2_regularization: 0.01, dropout_rate:0.2, optimizer: Adam, loss: binary_cross_entropy |
| OCEC without  breast, cervical cancers | Tensorflow/  custom model | number of encoder layers: 4, layer1_hidden_size: (48, 32, 16, 8),  number of classifier layers: 1, classifier_hidden_size: 16,  l2_regularization: 0.01, dropout_rate:0.2, optimizer: Adam, loss: binary_cross_entropy |
