# Supplementary material for: Patient-Level Cancer Prediction Models From a Nationwide Patient Cohort: Model Development and Validation
Source: JMIR Med Inform. 2021 Aug 30;9(8):e29807. doi: 10.2196/29807 (PMC8438609; doi:10.2196/29807)

**Multimedia Appendix 3. SHAP summary plot for each cancer.**

To help analyze the influence of each variable, the description for each variable and SHAP summary plot for each cancer are attached. In feature-specific plot in Figure S1-S6, each dot comes from an individual patient. The color of the dot means the feature value of an individual patient, and it means a higher value from blue to red. The position of the dot indicates the effect of cancer prediction. Dots to the right are used to increase the probability of cancer, and dots to the left are used to decrease the probability of cancer. Therefore, the red dots on the right and blue dots on the left means positive correlation the variable has with cancer prediction.

**Table S3. Feature name and description**

| Feature name | Feature description |
| --- | --- |
| SEX | Patient's gender (0: mail, 1: femail) |
| AGE | Patient's age |
| BMI | Body Mass Index |
| BP_HIGH | Systolic blood pressure |
| BP_LWST | Diastolic blood pressure |
| BLDS | Blood sugar before meals |
| TOT_CHOLE | Total cholesterol |
| HMG | Hemoglobin |
| OLIG_PROTE_CD | Urine protein level |
| SGOT_AST | (Serum GOT) AST |
| SGPT_ALT | (Serum GPT) ALT |
| GAMMA_GTP | Gamma GTP |
| SMK_STAT_TYPE_RSPS_CD | Smoking status (0:non-smoker, 1:past-smoker, 2: current smoker) |
| CUR_DSQTY_RSPS_CD | Amount of smoking per day (current) |
| SMK_TERM_RSPS_CD | Total smoking period |
| DRNK_HABIT_RSPS_CD | Number of drinks per week |
| TM1_DRKQTY_RSPS_CD | Drinking amount per time |
| EXERCI_FREQ_RSPS_CD | Number of workouts per week |
| FMLY_APOP_PATIENT_YN | Family history of stroke |
| FMLY_CANCER_PATIENT_YN | Family history of cancer |
| FMLY_DIABML_PATIENT_YN | Family history of diabetes |
| FMLY_HDISE_PATIENT_YN | Family history of heart disease |
| FMLY_HPRTS_PATIENT_YN | Family history of high blood pressure |
| HCHK_APOP_PMH_YN | (Self) Past history of stroke |
| HCHK_DIABML_PMH_YN | (Self) Past history of diabetes |
| HCHK_ETCDSE_PMH_YN | (Self) Past history of other diseases |
| HCHK_MDISE_PMH_YN | (Self) Past history of heart disease |
| HCHK_HPRTS_PMH_YN | (Self) Past history of high blood pressure |
| HCHK_PHSS_PMH_YN | (Self) Past history of pulmonary tuberculosis |

**Figure S1. SHAP summary plot for liver cancer.**


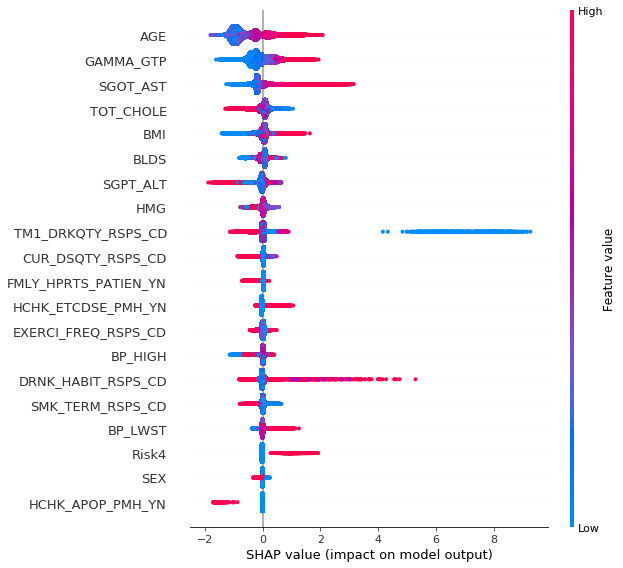


**Figure S2. SHAP summary plot for lung cancer.**


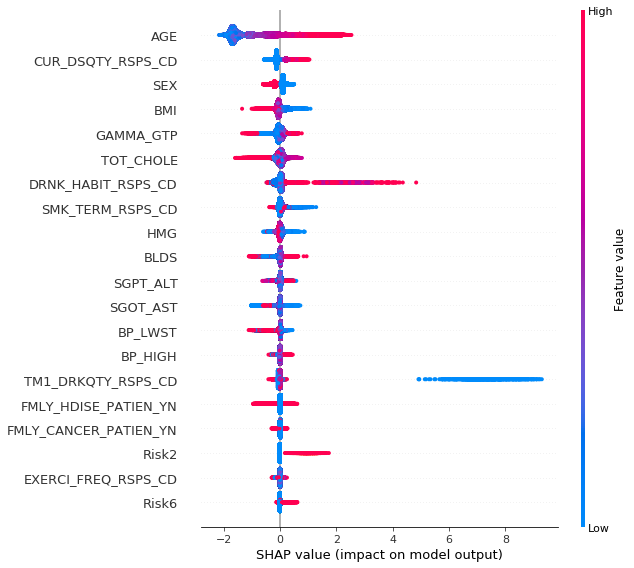


**Figure S3. SHAP summary plot for colorectal cancer.**


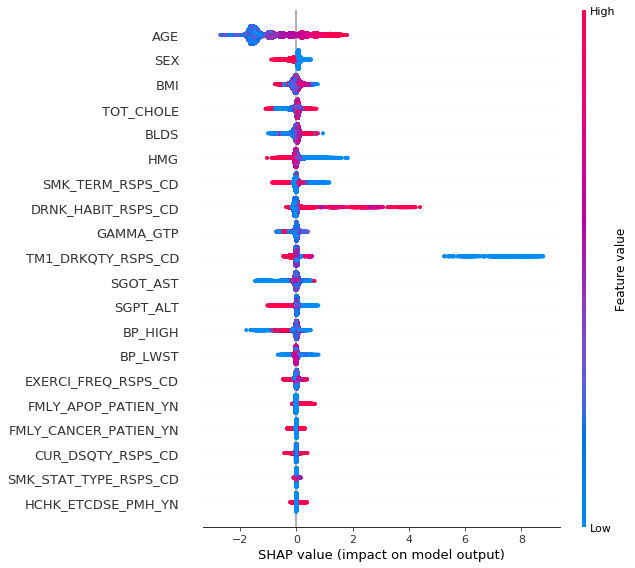


**Figure S4. SHAP summary plot for pancreatic cancer.**


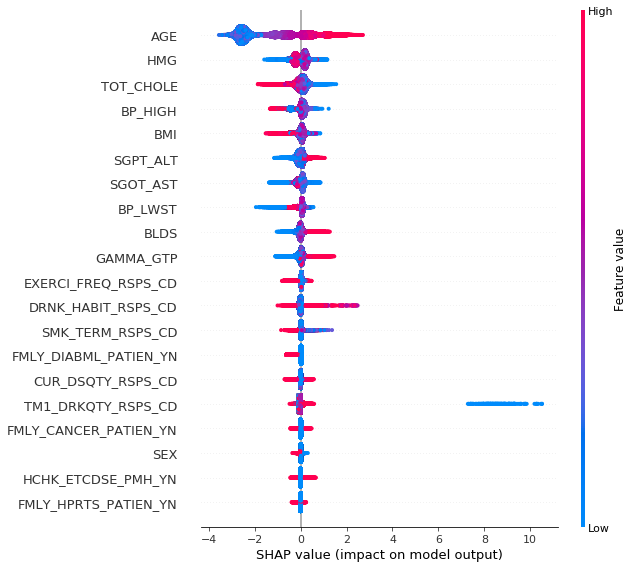


**Figure S5. SHAP summary plot for stomach cancer.**


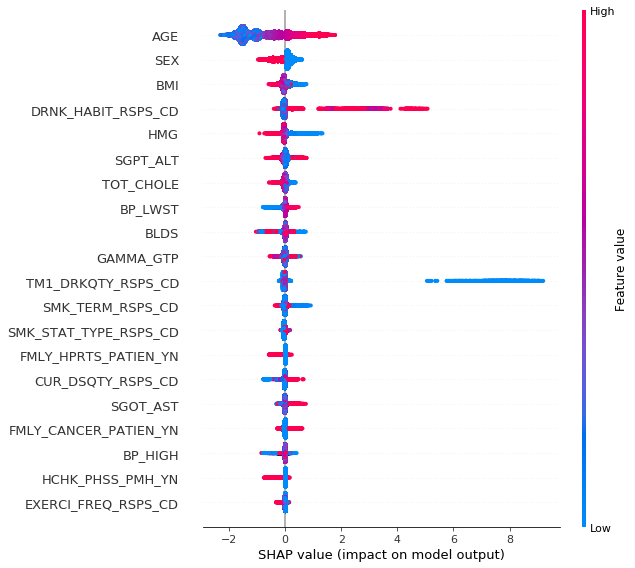


**Figure S6. SHAP summary plot for breast cancer.**


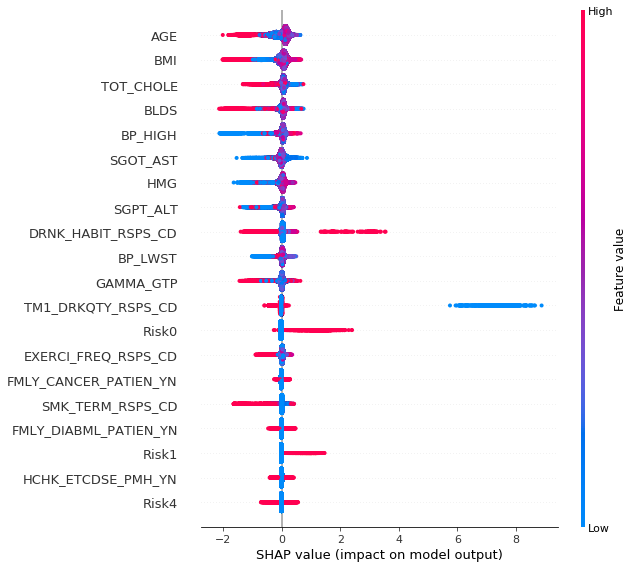


**Figure S7. SHAP summary plot for cervical cancer.**


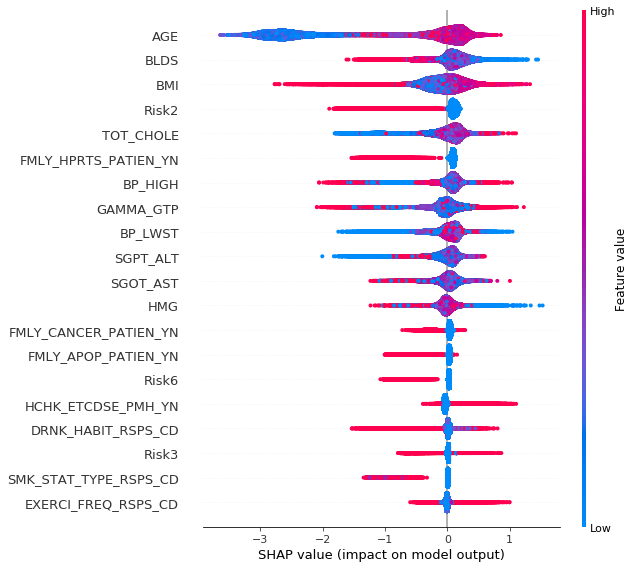

Supplement: Multimedia Appendix 3 [file medinform_v9i8e29807_app3.docx]
